# Recent Progress of Layered Double Hydroxide-Based Materials in Wastewater Treatment

**DOI:** 10.3390/ma16165723

**Published:** 2023-08-21

**Authors:** Yanli Fu, Xiaoqian Fu, Wen Song, Yanfei Li, Xuguang Li, Liangguo Yan

**Affiliations:** School of Water Conservancy and Environment, University of Jinan, Jinan 250022, China; f18766162009@163.com (Y.F.); fuxiaoqian02@163.com (X.F.); stu_songw@ujn.edu.cn (W.S.); stu_liyf@ujn.edu.cn (Y.L.); stu_lixg@ujn.edu.cn (X.L.)

**Keywords:** LDHs, coprecipitation, hydrothermal, adsorbent, catalyst, advanced oxidation processes, electrocatalyst

## Abstract

Layered double hydroxides (LDHs) can be used as catalysts and adsorbents due to their high stability, safety, and reusability. The preparation of modified LDHs mainly includes coprecipitation, hydrothermal, ion exchange, calcination recovery, and sol–gel methods. LDH-based materials have high anion exchange capacity, good thermal stability, and a large specific surface area, which can effectively adsorb and remove heavy metal ions, inorganic anions, organic pollutants, and oil pollutants from wastewater. Additionally, they are heterogeneous catalysts and have excellent catalytic effect in the Fenton system, persulfate-based advanced oxidation processes, and electrocatalytic system. This review ends with a discussion of the challenges and future trends of the application of LDHs in wastewater treatment.

## 1. Introduction

Hydrotalcite, hydrotalcite-like compounds, and their intercalation materials are collectively referred to as layered double hydroxides (LDHs), which have the chemical formula of [$M_{1-x}^{2+}$$M_{x}^{3+}$(OH)_2_]^x+^[(A^n−^)_x/n_·mH_2_O]^x−^ (M^2+^ for divalent metal cations, such as Mg^2+^, Co^2+^, etc., M^3+^ for trivalent metal cations, such as Fe^3+^, Al^3+^, etc., and A^n−^ for inorganic anions, such as NO_3_^−^, CO_3_^2−^, etc.). Compared with other layered materials, the type and ratio of metals in LDHs and the type of interlayer anions can be regulated to a certain extent; thus, various LDHs with different structures and functions are prepared [1]. It is possible to modulate or modify the composition or structure of LDHs according to different requirements to obtain LDH-based materials that meet different needs. Due to the advantages of easy synthesis, low toxicity, and chemical stability, LDHs are widely used in the fields of adsorption [2], catalytic chemistry [3], electrochemistry [4], photochemistry [5,6], etc. For example, LDHs and LDH-based materials can be used as adsorbents to remove heavy metal ions [7], refractory organics [8], and other pollutants from water because of their “ion-exchangeability”, “memory effect”, and other properties.

Among various water and wastewater treatment methods, adsorption and catalytic methods are commonly used because of their lower operating cost and higher treatment efficiency [9,10]. In addition, LDHs also have a broad application prospect in the field of water pollution treatment. To date, several review articles have discussed the application of LDHs and their composites in water treatment, including LDHs for the treatment of dyes, heavy metals, and anionic wastewater [11,12,13,14]. In recent years, lots of work has been conducted on the application of LDH-based materials in adsorption and catalysis, and few comprehensive reviews have been published [15,16]. 

To further explore the research progress of LDHs in the wastewater treatment process, the preparation and improvement of LDHs and their applications as adsorbents and catalysts are reviewed, which can provide a reference for the application of LDHs in wastewater treatment. The objectives of this work include the following: (1) to summarize the synthesis methods of properties of LDHs and their composites; (2) to review the research progress of adsorption and catalytic performance of LDH-based materials for the removal of inorganic and organic pollutants from wastewater; (3) to focus on the interaction mechanisms between LDHs and pollutants using adsorption and catalytic technologies. The challenges and future trends of the application of LDHs in wastewater treatment are also discussed at the end of this review.

## 2. Preparation and Modification of LDHs

LDHs have attracted much attention since its discovery of brucite, and the limited amount and purity of natural LDHs have made synthetic LDHs a hot spot for various applications due to their metal type and interlayer space tunability [17]. LDH can be classified as binary, ternary, and tetradentate LDHs according to the composition and number of metal elements. A binary LDH consists of two cations; a ternary one involves three cations, and the quaternary LDH contains four cations [18]. Chen et al. [19] prepared a binary LiAl-LDH using a one-step co-precipitation method to investigate the effect of interlayer anion of SO_4_^2−^ on the adsorption performance. Li et al. [20] synthesized a ternary NiCo_2_Mn-LDH for arsenate adsorption based on the M-O octahedral structure of Ni(II), Co(II), and Mn(III). Enkhtuvshin et al. [21] prepared a tetradentate NiFeAlCo-LDH using a MOF-derived electrochemical activation method. The overpotential of NiFeAlCo-LDH was as low as 220 mV at a current of 100 mA cm^−2^, which was caused by the oxygen intermediate that could synergize with neighboring metals.

LDH-based composites also have been studied in recent years. Zhang et al. [4] loaded CoAl-LDH onto biochar to remove heavy metals and PAHs by electrochemical activation of peroxymonosulfate (PMS). Li et al. [22] prepared flower-like nanosheets with Fe_2_O_3_/NiFe-LDH reaching a low overpotential of 220 mV at a high current density of 500 mA cm^−2^, in that Fe_2_O_3_ and NiFe-LDH exhibited extremely high catalytic activity in concert. Li et al. [23] deposited LDHs on the ZIF-67 surface along the nanofibers to obtain nanoflower-branch composites (CoNi-LDH@CNFs) with a rich cavity structure supported by electrospun nanofibers, which have excellent cathodic performance in microbial fuel cells (MFC) with a maximum power density of 1390.37 mW/m^2^. 

In addition, LDHs are of great interest because of their adjustable metal ions, multiple metal combinations, simple preparation methods, and high functionality. By controlling the nucleation and growth rate of metal salts, the morphology and structure of LDHs can be controlled, and the preparation method of LDHs with excellent properties is the focus [24]. The common synthesis approaches of modified LDHs mainly include coprecipitation, hydrothermal, ion exchange, calcination recovery, and sol–gel methods.

### 2.1. Coprecipitation Method

**Figure 1:**
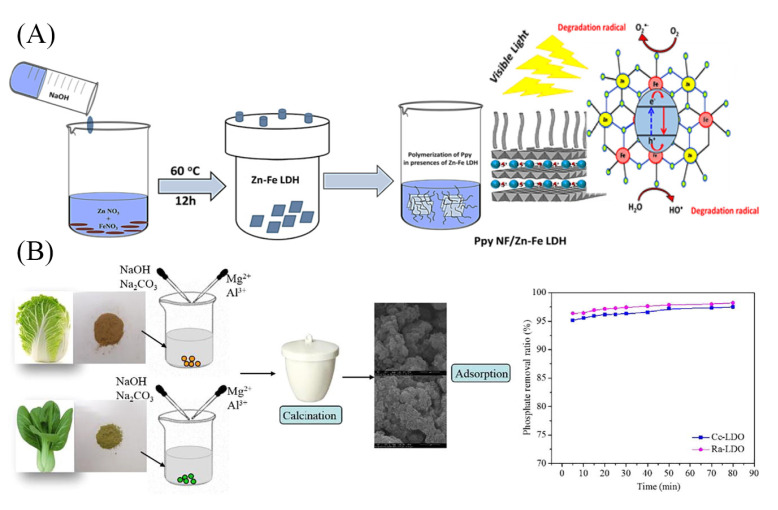
Schematic diagram of the synthesis of (**A**) PPy NF@ZnFe LDH [27], (**B**) Biochar/MgAl-LDO by coprecipitation method [33]. Reprinted with permission from Elsevier.

The coprecipitation method is commonly used for the preparation of various LDHs and is also applied in the synthesis of LDH-based composites. Generally, the divalent and trivalent cations that constitute the LDHs laminate were mixed as a salt solution, and the target materials were added to the salt solution in a certain proportion. Then, the pH was adjusted to a constant value with Na_2_CO_3_ or NaOH solution under stirring at a certain temperature. Finally, the solids were washed and dried to obtain the LDH-based composites [25]. Lu et al. [26] formed a novel Fe_3_O_4_@MgAl-LDH on the surface of Fe_3_O_4_ microspheres. The dense negative charge on the surface of Fe_3_O_4_ microspheres and the electrostatic interaction between cations in the metal salt solution were used to form LDHs on the surface of Fe_3_O_4_. During the preparation process, the pH of the Fe_3_O_4_ suspension was adjusted to 10 with salt and alkaline solutions to make the surface of Fe_3_O_4_ microspheres with a partial negative charge. When adjusting the pH, vigorous stirring was required to make the Mg^2+^ and Al^3+^ cations adsorb quickly on the surface of Fe_3_O_4_ by electrostatic attraction. Mohamed et al. [27] prepared ZnFe-NO_3_ LDH with n(Zn)/n(Fe) of 2.0 by coprecipitation method. Then, ZnFe-NO_3_ LDH was mixed with chloroform containing pyrrole and stirred for 1 h to form a solution, and the oxidants, by mixing hydrochloric acid with potassium persulfate, were transferred to this solution to form a black–brown precipitate. The solids were washed and dried with ethanol and distilled water at room temperature to obtain PPy NF@ZnFe-LDH. The above process is shown in Figure 1A. The synthesized ZnFe-NO_3_ LDH exhibited plate-like or sheet-like particles in the form of separated particles or agglomerated particles. After the formation of PPy NF@ZnFe-LDH nanocomposites, the ZnFe-NO_3_ LDH particles were completely covered by a layer of fibrous PPy with a smooth surface and uniform diameter. The characterization image of polymer fibers reflected that the interlocking between fibers formed a porous network structure, giving the final product a higher surface area and pore volume and thus facilitating the performance as an adsorbent or catalyst carrier. 

Yan’s research group [28,29,30,31,32,33], prepared a series of LDHs and their composites using the coprecipitation method by changing the type of metal salts, the composition of alkali solution, and adding Fe_3_O_4_, biochar, and other substrates. Characterization results, such as X-ray diffraction (XRD), Fourier transform infrared (FTIR) spectroscopy, and scanning electron microscopy (SEM), showed that the as-prepared LDHs were well crystalline with a typical hexagonal layered structure [28]. The intercalated LDHs obtained by dissolving EDTA, L-cysteine, and other intercalating agents in an alkaline solution not only retained the crystal structure of LDHs but also brought in functional groups, which improved the ability of intercalated LDHs to remove pollutants from water [29,30]. The LDH-based hybrids with magnetic core-shell structures were also synthesized using Fe_3_O_4_ as a substrate. Fe_3_O_4_@MAl-LDHs (M = Mg^2+^, Zn^2+^, Ni^2+^) possessed superparamagnetism as well as the crystal structure and properties of LDHs, which enabled the rapid solid–liquid separation of the material from the mixture after reaction and facilitated material recovery [29,31]. The biochar/MgAl layered double oxide (LDO) was prepared from vegetable biochar and calcination, and the schematic diagram is shown in Figure 1B. The biochar distributed as a contributing matrix in the LDH interlayer after pyrolysis significantly increased the maximum adsorption capacity of both heavy metals and anionic pollutants in the water [33]. 

In order to obtain a good crystalline structure of the LDHs prepared by coprecipitation, the resulting products need to be aged for a long time. In addition, the particle size of LDHs obtained by the coprecipitation method tends to be unevenly distributed [34].

### 2.2. Hydrothermal Method

The hydrothermal method is a crystallization process under a higher temperature and pressure for a period of time; then, the solid is filtered, washed, and dried to produce LDHs. The LDHs prepared by this method have the characteristics of high purity, good dispersion, uniform particles, and controllable morphology than that of the coprecipitation method [35]. The high-temperature hydrothermal treatment allowed for the growth of LDH microcrystals with regular shapes and the controlled LDHs with different morphologies and sizes could be achieved by controlling the reaction temperature and reaction time [36]. Liang et al. [37] prepared the MgAl-LDHs with a stoichiometric ratio by reacting magnesium nitrate as well as aluminum nitrate salt solutions in a mixed solution of formamide and sodium hydroxide at a temperature of 60 °C for 24 h. During the hydrothermal process, the reagents with slow hydrolysis, such as urea, are often added to promote the crystallization in the 2D direction and the formation of stable ultrathin structure of LDHs [38]. Rathee et al. [39] synthesized NiFeTi-LDH in different proportions by mixing nickel nitrate, iron nitrate, titanium tetrachloride, and urea and hydrothermally aged in an autoclave at 160 °C for two days. The composites showed a distinct lamellar structure. 

**Figure 2:**
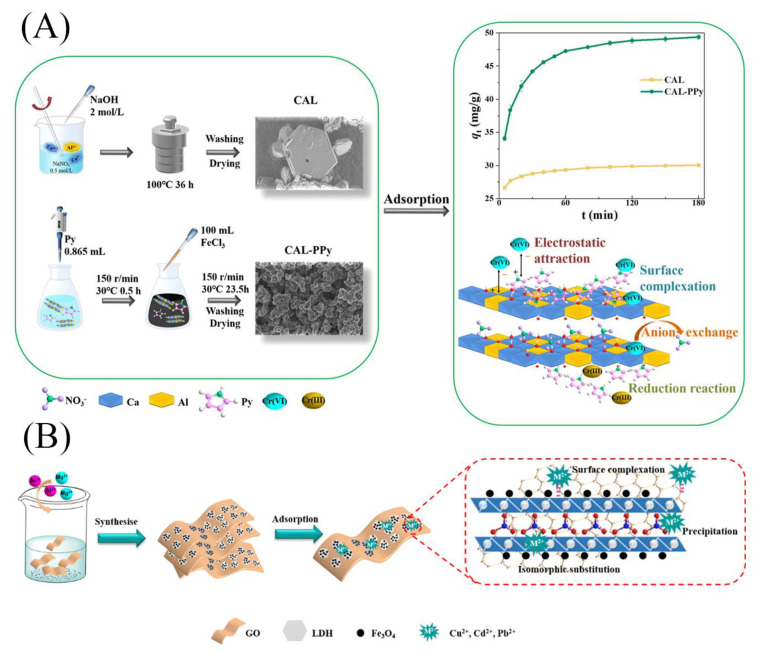
Schematic diagram of the synthesis of (**A**) CAL and CAL-PPy [40] and (**B**) magnetic MgAl-LDH/graphene oxide composite [41]. Reprinted with permission from Elsevier.

Some LDH-based composites were prepared using the hydrothermal method by changing the type of metal salts, the composition of the alkali solution, and the addition of Fe_3_O_4_ [40,41]. For example, the CaAl-LDH (CAL) was prepared by hydrothermal method and had typical LDH peaks, good crystallinity, and hexagonal layered structure, which could efficiently and rapidly remove hexavalent chromium (Cr(VI)) from the water [40]. To further increase the capacity, CaAl-LDH was combined with polypyrrole (PPy) to obtain (CAL-PPy) (Figure 2A) [40]. The characterization results showed that PPy was coated on the surface of hexagonal CAL. The addition of PPy introduced many amine functional groups, which improved the adsorption efficiency. To reduce the cost of composite materials and increase their reusability, MgAl-LDH was assembled onto graphene oxide and its magnetic product containing Fe_3_O_4_ [41]. The preparation process is shown in Figure 2B. The composites had abundant functional groups and large specific surface area and could effectively remove heavy metals under neutral and weak alkaline conditions. Moreover, magnetic nanomaterials could achieve simple and effective solid–liquid separation under a magnetic field. However, the preparation process of LDHs using the hydrothermal method is more energy-intensive and generally requires specific reaction vessels.

### 2.3. Ion Exchange Method

**Figure 3:**
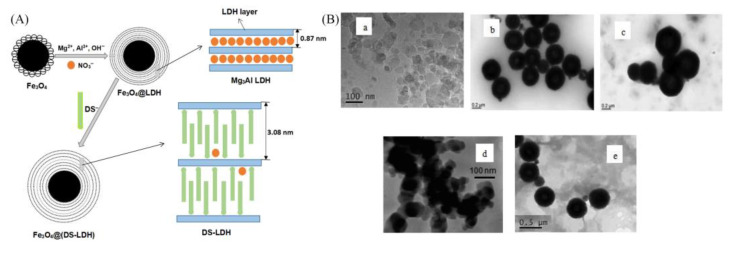
(**A**) Schematic diagram for the preparation of Fe_3_O_4_@(DS-LDH) [43]. (**B**) TEM photos of (**a**) Mg_3_Al LDH, (**b**) Fe_3_O_4_, (**c**) Fe_3_O_4_@LDH, (**d**) DS-LDH, and (**e**) Fe_3_O_4_@ (DS-LDH) [43]. Reprinted with permission from Elsevier.

The ion exchange method is based on the characteristics of LDHs with anion exchange. Usually, synthetic LDHs are used as precursors, and the anions of the target product are exchanged with the interlayer anions to produce the desired LDHs. The ion exchange method allows for the synthesized LDHs with various interlayer anions, and the loading amounts need to be further improved. González et al. [42] synthesized MgAl-LDH-Cl with Cl^−^ as the interlayer anion by coprecipitation method, and then, the humic acid (HA) was added to synthesize LDH-HA adsorbent by ion exchange. It was found that the functional groups of HA had a high affinity for metal cations, and LDH-HA could remove Cu^2+^, Pb^2+^, and Cd^2+^ efficiently and rapidly. Li et al. [43] synthesized Fe_3_O_4_@(DS-LDH) by coprecipitation and ion exchange methods and used blue and methyl orange in water for the removal of methylene. The Fe_3_O_4_ powder was firstly prepared by solvothermal method; then, Fe_3_O_4_@MgAl-LDH with flaky core-shell structure was synthesized by coprecipitation method, and finally, Fe_3_O_4_@(DS-LDH) with SDS intercalation was prepared by ion exchange method (Figure 3A). The transmission electron microscopy image in Figure 3B showed that the LDH particles were hexagonal nanosheets of 50–100 nm. The shape of Fe_3_O_4_ particles was close to spherical. The morphology of DS-LDH particles was similar to that of Mg_3_Al-LDH, and the size of DS-LDH particles was slightly increased. In addition, DS-LDH particles were significantly aggregated, which may be due to the enhanced hydrophobicity of the particles. The surfaces of Fe_3_O_4_@LDH and Fe_3_O_4_@(DS-LDH) were rougher than the pristine Fe_3_O_4_, and a thin layer of LDH (or DS-LDH) was found to cover Fe_3_O_4_, indicating that Fe_3_O_4_@LDH and Fe_3_O_4_@(DS-LDH) formed a core-shell structure. 

### 2.4. Calcination Recovery Method

**Figure 4:**
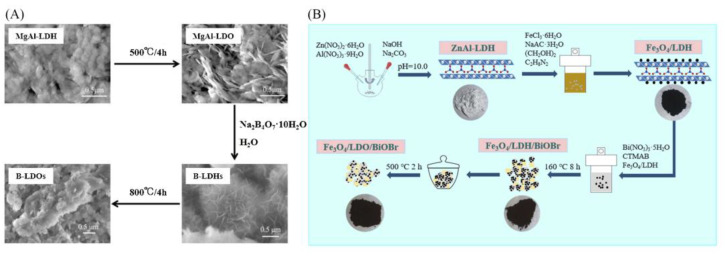
Schematic diagram of the preparation of (**A**) B-LDO [44] and (**B**) Fe_3_O_4_/LDO/BiOBr-x [45]. Reprinted with permission from Elsevier.

The calcination recovery method is based on the special “memory effect” of LDHs; i.e., the LDHs precursors are firstly heat-treated at a certain temperature to obtain roasting products of LDOs, and then, it is added to a solution containing target anions to restore the original lamellar structure to obtain a new LDHs. LDHs are limited in their application as adsorbents for anionic pollutants because the interlayer carbonate anions are not easily exchanged with other anions, and most of the prepared classical LDHs are not hierarchically porous nanostructures. To solve this problem, Jia et al. [44] roasted MgAl-LDH at 500 °C for 4 h to obtain the precursor MgAl-LDO, reduced MgAl-LDO to obtain the borate intercalated MgAl-LDH (B-LDH), and finally, roasted at 800 °C for 4 h to obtain B-LDO (Figure 4A). The synthesized B-LDO had higher adsorption efficiency, good reusability, and stability and were more suitable for wastewater treatment. Li et al. [45] prepared ZnAl-LDH by coprecipitation method, and then, Fe_3_O_4_/LDH was synthesized by solvothermal method at 160 °C for 8 h. Finally, the obtained catalyst was calcined at 500 °C for 2 h to synthesize Fe_3_O_4_/LDO/BiOBr composite for efficient photoreduction in a high concentration of Cr(VI) (Figure 4B). A magnetic recycling experiment revealed that Fe_3_O_4_/LDO/BiOBr-1.5 can be easily reused for 4 cycles. 

It is worth noting that the calcination recovery method requires higher temperatures and higher energy consumption and may produce harmful gases during the calcination process.

### 2.5. Sol–Gel Method

Sol–gel method uses organometallic salts through a sol–gel transformation process [46,47]. It can control the morphology and size of the particle well, and then, the phase separation can be controlled by the hydrolytic condensation reaction of the alcohol salt [48]. Chubar et al. [49] investigated the synthesis of MgAl-LDH via sol–gel method with and without alcohol salts. The adsorption effect of the alkoxide-free sol–gel (Mg-Al-AFSG) is better than the alkoxide sol–gel (Mg-Al-ASG). This is because the interlayer carbonate ions of Mg-Al-AFSG are involved in the removal of anions, while the tightly bound bidentate coordination of Mg-Al-ASG leads to poor adsorption without the involvement of carbonate. The commonly used chemicals in sol–gel method are metal alkoxides, while inorganic salts are used to achieve more economical and environmental-friendly materials. Valeikiene et al. [50] studied the reconstitution properties of Mg_2−x_M_x_/Al_1_-LDH (M = Ca, Sr, Ba) prepared by sol–gel method. The results show that the microstructure of LDHs produced by sol–gel method is basically consistent with that of LDHs. Except for the adsorption performance, the stability of the adsorbent also plays an important role. Luo et al. [51] used the phase-separated sol–gel method to synthesize the nanocomposite of FeLiAl-LDH and FeOOH. The Fe(III) doping and FeOOH nanocomposite reduced the dissolution of LiAl-LDH in the granular nanocomposite and did not negatively affect the adsorption capacity.

## 3. Adsorptive Removal of Pollutants by LDH-Based Materials

There are many technical methods for wastewater treatment, among which the adsorption method has a good application prospect which has the advantages of simple operation, high efficiency, low cost, and reusability as an adsorbent. In recent years, lots of low-cost adsorbents, including natural clay minerals, industrial by-products, chitosan, organic composite materials, and biological adsorbents, have been prepared and used to remove different pollutants from water [52,53,54]. Among them, LDH-based materials are widely concerned as adsorbents because of their large specific surface area, low cost, easy synthesis, and reusability, and are expected to become promising new adsorptive materials.

### 3.1. Inorganic Pollutants

#### 3.1.1. Heavy Metals

**Figure 5:**
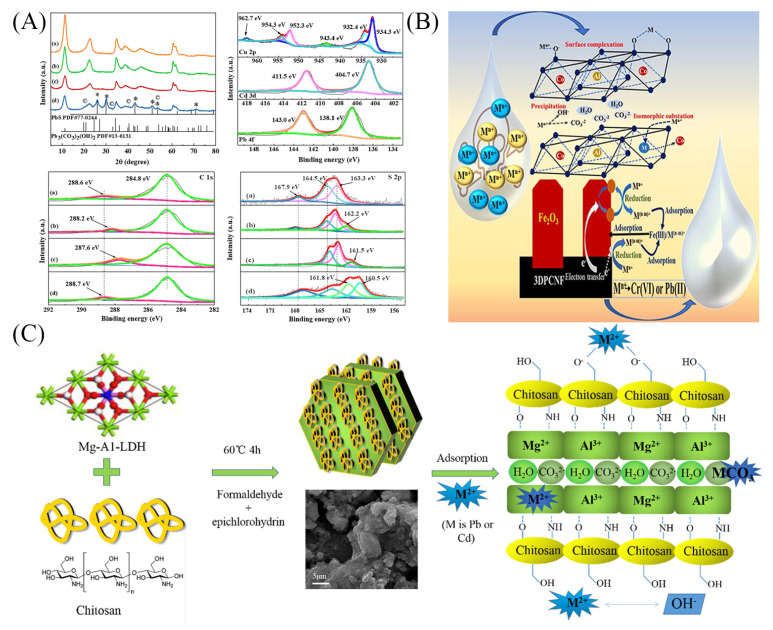
(**A**) XRD and XPS spectra of Pb 4f/Cd 3d/Cu 2p, C 1s and S 2p of MgAl-Cys-LDH before adsorption (a) and after adsorption of Cu(II) (b), Cd(II) (c) and Pb(II) (d) [29]. Schematic diagram of the mechanisms of heavy metal adsorption by (**B**) CS-LDH [60] and (**C**) Co-Al-LDH@Fe_2_O_3_/3DPCNF [63]. Reprinted with permission from Elsevier.

The application of LDHs in the adsorption of cationic heavy metals has received increasing attention [15,36]. Special functional materials can be obtained that have the advantages of both the interlayer anion and the main body of the LDHs [55]. Therefore, the adsorption capacity and selectivity of heavy metals can be improved by the functional ligands of the LDHs [55,56]. As can be seen from Table 1, a variety of modified LDH materials were synthesized to remove Cd^2+^, Pb^2+^, Cu^2+^, and Cr^6+^ in water [28,29,30,40,41,57,58,59,60,61]. The adsorption performance of LDHs for heavy metal ions was investigated in detail by optimizing the experimental conditions, such as adsorbent dosage, oscillation time, and solution pH, and by studying the adsorption kinetics, isotherms, and recycling properties. The mechanisms were also conducted by integrating spectroscopic characterizations and solution chemistry. Zhang et al. [29] used a coprecipitation method to synthesize L-cysteine intercalated MgAl-LDH, thereby introducing complexing groups, such as carboxyl, sulfhydryl, and amino groups, and then achieving efficient removal of heavy metals of Cu^2+^, Pb^2+^, and Cd^2+^ through hydroxide or sulfide precipitation, complexation of surface-rich groups, and isomorphous replacement of LDH (Figure 5A). Lyu et al. [60] synthesized CS-LDH (chitosan-modified MgAl-LDH), which was rich in surface functional groups, such as amino, hydroxyl, and amide groups, and had a loose and porous laminar structure. The adsorption process for Pb^2+^ quickly reached an equilibrium state within 10 min, and that of Cd^2+^ was 60 min. The adsorption capacities of CS-LDH for Pb^2+^ and Cd^2+^ were significantly larger than those of CS and MgAl-LDH and reached 333.3 mg/g and 140.8 mg/g, respectively. The adsorption mechanism was chemical precipitation, surface complexation, and isomorphous replacement (Figure 5B). The above works indicated that the adsorption of cationic heavy metals was mainly through the formation of surface hydroxide precipitation, complexation with functional groups, and isomorphous replacement of LDHs.

**Table 1:** Adsorptive removal of heavy metal ions by LDHs.

Adsorbent	Heavy Metal Ions	Adsorption Conditions			Theoretical Adsorption Capacity (mg/g)	Adsorption Mechanism	Ref.
		Adsorbent Dosage	pH	Oscillation Time (min)			
MgAl-CO_3_-LDH, Fe_3_O_4_/MgAl-CO_3_-LDH	Cd^2+^ Cd^2+^	0.08 g	4	60 300	61.40~70.20 45.60~54.70	precipitation, surface adsorption, surface complexation	[28]
MgAl-Cys-LDH	Cu^2+^ Pb^2+^ Cd^2+^	0.05 g	4	90 180 10	58.07 186.20 93.11	precipitation, surface complexation, isomorphous replacement	[29]
Fe_3_O_4_/LDH-AM	Cd^2+^ Pb^2+^ Cu^2+^	0.05 g	5~6	240 180 240	74.06 266.60 64.66	surface complexation, precipitation	[58]
Magnetic MgAl-LDO/carbon	Cd^2+^ Pb^2+^ Cu^2+^		6	90 120 400	386.10 359.70 192.70	precipitation, surface complexation, electrostatic attraction	[59]
CS/MgAl-LDH	Pb^2+^ Cu^2+^	0.05 g	6	10 60	333.30 140.80	precipitation, surface complexation, isomorphous replacement	[60]
CaAl-LDH	Cu^2+^ Cd^2+^	0.01 g	5/5.8		381.90 1035.40	precipitation, isomorphous replacement, surface complexation	[57]
GO/LDH Fe_3_O_4_@GO/LDH	Cu^2+^ Cd^2+^ Pb^2+^	20 mg		240	89.26~80.72 76.67~70.26 226.98~213.96	surface complexation, precipitation, isomorphous replacement	[41]
LDH-EDTA-AM	Cr^6+^			30	48.47	electrostatic attraction, surface complexation, ion exchange, reduction	[30]
Fe_3_O_4_-ZnAl-LDH/TiO_2_	Cr^6+^	0.02 g/L	3	480		electrostatic attraction, ion exchange, photoreduction	[61]
CaAl-LDH CAL-PPy	Cr^6+^	0.03 g			34.06 66.14	electrostatic attraction, surface complexation, anion exchange, reduction	[40]

For the heavy metals presented as anions, in addition to the above mechanisms, they could be adsorbed by LDHs via electrostatic attraction of positive surface charges and interlayer anions exchange [62]. As for Cr^6+^, there was a simultaneous adsorption-reduction coupling mechanism in which the electron-giving groups of LDHs reduced the adsorbed Cr^6+^ to Cr^3+^ and then adsorbed Cr^3+^ by complexation and isomorphous replacement [30]. This depended on some factors, such as the divalent and trivalent laminate ions of the prepared LDHs, the type of interlayer anions, and the conditions of the adsorption experiments. Yang et al. [40] prepared CAL and CAL-Ppy by hydrothermal and in situ polymerization methods, respectively. The addition of Ppy introduced many amine functional groups, increased the number of active sites, and improved the efficiency of CAL in the removal of Cr^6+^. The main removal mechanisms included electrostatic attraction, surface complexation, anion exchange, and reduction to less toxic Cr^3+^. Poudel et al. [63] prepared CoAl-LDH containing hematite (α-Fe_2_O_3_) @3D porous carbon nanofibers (Co-Al-LDH@Fe_2_O_3_/3DPCNF) by hydrothermal method, which had a maximum adsorption capacity of 400.40 mg/g for Cr^6+^. The increased specific surface area, ultra-high hydrophilicity, high affinity for metal ions, and three-dimensional cross-network nanostructure are the main reasons for the larger adsorption capacity. The mechanisms mainly included surface complexation, ion exchange, and reduction (Figure 5C). In addition, after 10 cycles, the composite was able to remove about 60% of the metal ions.

#### 3.1.2. Inorganic Anions

Sulfate, phosphate, and nitrate ions in wastewater are common pollutants. Among them, sulfate ions are generally removed by adding solid calcium hydroxide (Ca(OH)_2_) to precipitate gypsum, but the formed precipitate usually takes much time to settle, and the limited solubility of Ca(OH)_2_ makes the removal of sulfate unsatisfactory. To improve the removal efficiency of sulfate ions, Maziarz et al. [64] investigated the effect of LDHs addition, Ca(OH)_2_ addition, a mixture of Ca(OH)_2_ and LDHs, and the use of LDHs after precipitation with Ca(OH)_2_ on the removal of sulfate from water. It was found that the use of a mixture of Ca(OH)_2_ and LDHs resulted in simultaneous precipitation and adsorption of sulfate with higher removal efficiency. In addition, the application of the mixture also resulted in a significant reduction in sludge volume compared to the experiments using only Ca(OH)_2_. Zhang et al. [33] loaded MgAl-LDH onto cabbage and rape biomass by coprecipitation and followed by oxygen-limited calcination to obtain two biochar/LDO composites with large specific surface areas and abundant surface functional groups. At a dosage of only 0.05 g of biochar/LDOs, the removal process of 50 mg/L phosphate in the pH range of 2–10 was rapid, removing more than 92% of phosphate in 5 min. Based on FTIR, XPS, and zeta potential analysis, the adsorption mechanisms included the memory effect of LDHs, electrostatic attraction, surface complexation, and interlayer anion exchange. 

Radioactive waste from nuclear power endangers the existence of living things. Adsorption is one of the main treatment methods for radioactive wastewater. Yang et al. [65] synthesized a composite of MgCo-LDHs and dendritic fibrous nanosilica (DFNS) via an interface-constrained strategy. The DFNS@Mg-Co LDH showed high uranium (U(VI)) uptake capacity (1143 mg g^−1^) at pH = 3 and *C*_0_ = 598.7 mg L^−1^, which was about 4.8-fold higher than that of pristine DFNS. Yang et al. [66] prepared a MgAl-LDH intercalated with salicylaldoxime (SA-LDH), which exhibited excellent U(VI) capture performance. The tremendous maximum sorption capacity was 502 mg·g^−1^, surpassing most known sorbents. The interaction mechanisms included complexation (UO_2_^2+^ with SA^−^ and/or CO_3_^2−^), ion exchange, and precipitation. 

### 3.2. Organic Pollutants

#### 3.2.1. Dyes

Dyes are widely used in industry and can prevent sunlight and oxygen from entering the water when they are discharged into water bodies [67]. Because of the anion exchange capacity, memory effect, and large specific surface area, LDHs have shown excellent adsorption performance in the treatment of printing and dyeing wastewater. The adsorption mechanisms of LDHs for dyes can usually be divided into three main categories: (1) electrostatic attraction, whereby the hydrophilicity and hydrophobicity of LDH surface can be controlled and used to adsorb different types of dyes; (2) ion exchange, whereby the anions in the dye molecules are exchanged with the interlayer anions of LDH; (3) complexation, whereby the dye molecules complexed or chemical bonded with functional groups of LDHs to achieve efficiency removal of dye molecules from wastewater.

A series of functional LDH-based materials for removing dyes from water were synthesized [30,31]. To better investigate the removal of dyes from combined contaminants, Li et al. [30] prepared LDH-EDTA-AM (EDTA-intercalated MgAl-LDH crosslinked with acrylamide) composite. It was found that the adsorption of congo red (CR) by LDH-EDTA-AM did not affect by Cr^6+^, while the removal of Cr^6+^ was promoted by CR. The main adsorption mechanisms include electrostatic attraction, surface complexation, and anion exchange. Soltani et al. [68] synthesized LDH/MOF composite for the treatment of a reactive dye Orange II. The higher specific surface area (1282 m^2^ g^−1^) improved the adsorption for orange II, and the maximum adsorption amount reached 1173 mg/g. Mallakpour et al. [69] prepared the nanocomposite beads containing chitosan (Chi), tannic acid (TA), LDH, and mixed metal oxides (MMO). The beads were applied for the simultaneous removal of three reactive dyes, and the maximum adsorption capacities of Chi/TA@LDH and Chi/TA@MMO beads were between 257 and 483 mg g^−1^.

#### 3.2.2. Oil

Oily wastewater typically contains dispersed oil, suspended particles, fats, greases, hydrocarbons, and petroleum fractions [70]. The oil forms an insoluble film on the surface of the water, preventing aeration and natural lighting of the water body. In addition, it can contaminate groundwater, aquifers, and drinking water due to the infiltration of contaminants, causing adverse effects and ecological problems [71].

Due to the persistence of oily wastewater in the aquatic environment, conventional biological processes cannot remove it quickly, which often hinders the treatment of industrial-scale systems [72]. With in situ feasibility, cost-effectiveness, and environmental friendliness, adsorption is one of the most promising technologies for efficient oil–water separation [73,74], and LDHs are considered to be potential adsorbents. Menino et al. [75] used sodium dodecyl sulfate (SDS) and Fe_3_O_4_ to change the properties of LDHs and prepared a hydrophobic magnetic adsorbent (LDH-MSDS) with a maximum adsorption capacity of 659.90 mg g^−1^ for oil. The results showed that the hydrophobic surface combined with the superwetting and strong adhesion of Fe_3_O_4_ were favorable for the selective adsorption of oil-containing wastewater.

As is well known, superhydrophobic/superoleophilic materials can be used to remove oil from oil–water mixtures. However, the filtration process often results in the blockage of membrane pores by oil droplets [76]. Then, the development of superhydrophilic/superoleophobic films with anti-fouling properties has received much attention [68,69]. Cui et al. [77] prepared the grass-like composite membranes (NiCo-LDH/PVDF) by growing NiCo-LDH on the surface of polydopamine-modified polyvinylidene fluoride (PVDF) membrane using the hydrothermal method. The membrane exhibited a superhydrophilic/superoleophobic surface with good antifouling properties and could be recycled over a long period. Xie et al. [78] prepared a new active antifouling carbon cloth@NiCo-LDH/Ag (CC@LDH/Ag) membrane. The results showed that the CC@LDH/Ag membrane had superhydrophilic/superoleophobic performance and high separation efficiency for different oil–water mixtures. In addition, CC@LDH/Ag membrane also exhibited good self-cleaning ability.

#### 3.2.3. Persistent Organic Pollutants (POPs)

Contamination of groundwater by pesticides in modern agriculture systems is a matter of big concern and needs to be treated urgently. Lartey-Young and Ma [79] investigated the adsorption performance of CuZnFe-LDH and the composite with bamboo biochar (LDHBC) for atrazine removal from an aqueous solution. The removal of atrazine was up to 74.8%, and the adsorption mechanisms could be determined by π-π interactions occurring at the interfaces by hydrogen bonding and pore-filling effect. Mourid et al. [80] prepared the Zn_2_Al-LDH by coprecipitation at a constant pH, which has been used to remove 2,4,5-trichlorophenoxyacetic acid (2,4,5-T) herbicide. The removal rate reached 96% for an optimal molar ratio 2,4,5-T/Cl = 0.5. The partial or total release of the herbicide was observed to depend on the composition of the desorbing solution. This suggested the possibility of LDH recycling and confirmed its effectiveness in eliminating this type of pollutant from water.

Per- and polyfluoroalkyl substances (PFAS) are widely used in a variety of industries and have a high chemical stability because of the high C-F bond energies, which makes them particularly persistent in environmental media [81]. Removal of PFAS from contaminated water by adsorption is considered to be an effective and simple method. Chen et al. [82] prepared an LDH with the metal composition of Cu(II)Mg(II)Fe(III) for the fast adsorption of perfluorooctane sulfonate (PFOS) and perfluorooctanoate (PFOA). A total of 84% of PFOS and 48% of PFOA were adsorbed in the first minutes when contacted with 0.1 g/L of suspended µm-sized LDH particles. Hydrophobic interactions were primarily responsible for the adsorption of these compounds in accordance with the different adsorption affinities of long-chain and short-chain perfluorinated carboxylic acids. It was previously demonstrated that the modification of minerals with organic functional groups enhanced the adsorption of organic pollutants [14]. Min et al. [83] introduced organic functional groups to the ZnAl-LDH by covalent bonding through a post-grafting process. The organically functionalized LDHs were efficient for PFOA adsorption. This resulted from the synergy of the positively charged structural layers of LDHs that provided strong electrostatic interactions and the modified organic functional groups that provided enhanced hydrophobic interactions to capture PFOA.

### 3.3. Other Pollutants

Except for the above-mentioned inorganic and organic pollutants, microplastics (MPs) are becoming an intractable environmental issue due to their non-biodegradable nature and wide spreading. Tiwari et al. [84] synthesized the nanostructured ZnAl-LDH via the co-precipitation method and used it for the adsorption of nano-scale plastic debris (NPDs) from an aqueous solution. The negatively charged NPDs were adsorbed on ZnAl-LDH by electrostatic interaction, and 96% of NPDs was removed in deionized water. The removal efficiency was found to be 100% at a pH of 4 and declined to 37% at a pH of 9 due to the increased competitive binding and destabilization of LDH under alkaline conditions.

To address the challenge of eliminating MPs in the case of acidic or alkaline wastewater, Peng et al. [85] engineered a high-performance capture agent for polystyrene (PS) in a wide pH range, i.e., three-dimensional graphene-like carbon-assembled LDO (G@LDO). In virtue of the mutual protection effect of graphene-like carbon and LDO, G@LDO featured the preeminent acid and base resistance for PS removal. The removal efficiency of PS was ≥80% at pH = 3–11, and nearly 60% of PS was removed at pH of 1 and 13. Furthermore, the removal mechanisms were hydrogen bond and complexation from LDH recovered from LDO, as well as π-π/p-π interactions of carbon-containing sulfur. 

## 4. Application of LDHs in Advanced Oxidation Processes

The adsorption process only removes pollutants from the aqueous solution to the adsorbent surface, and the adsorbed contaminants are not degraded [18]. Therefore, there is an urgent need for an efficient way to degrade pollutants. In recent years, advanced oxidation processed (AOPs) have gained much attention for their ability to completely degrade pollutants to CO_2_ and H_2_O [86]. It mainly includes Fenton, persulfate, and electrochemical processes.

### 4.1. Fenton-like Reaction

**Figure 6:**
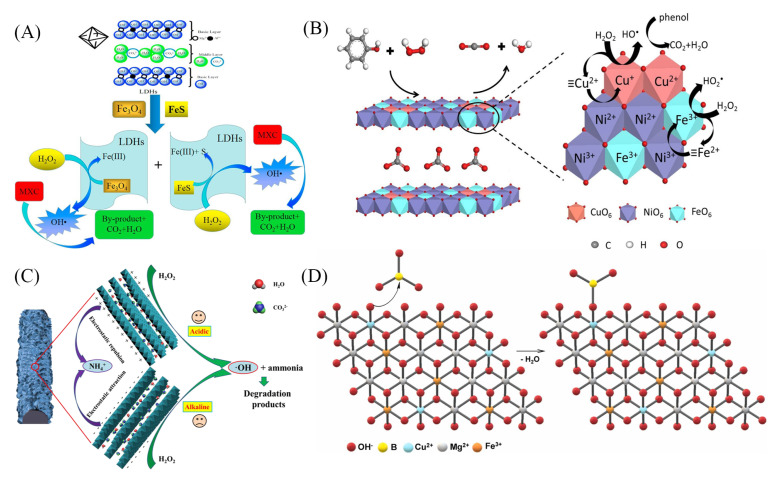
(**A**) Schematic diagram of Fenton reaction catalyzed by FeS@LDH and Fe_3_O_4_@LDH [87]. (**B**) Reaction mechanism of phenol degradation by CuNiFe-LDH [90]. (**C**) Illustration of the mechanism of ammonia nitrogen degradation by 2-CuAl/CF-LDH [92]. (**D**) Surface reaction between CuMgFe-B(OH)_4_ and B(OH)_3_ [93]. Reprinted with permission from Elsevier.

Fenton reaction is a process that uses the catalytic reaction of H_2_O_2_ and Fe^2+^ to rapidly generate highly reactive ^•^OH radicals, which is widely used in the wastewater treatment [87]. The generation of ^•^OH in the Fenton reaction is closely related to iron ions, and it has been shown that the incorporation of FeS and Fe_3_O_4_ nanoparticles significantly enhanced the coupling of Fe^2+^ and S^−^ and the synergistic effect of FeS on the Fenton process accelerated the dechlorination and degradation of organochlorine compounds [88]. Furthermore, Huang et al. [87] obtained FeS@LDH and Fe_3_O_4_@LDH composites to catalyze the Fenton reaction for the degradation of methoxychlor. In the catalytic degradation process of Fe_3_O_4_@LDH, the reductive dechlorination, cleavage, and oxidation of methoxychlor occurred; while using FeS@LDH and Fe_3_O_4_@LDH hybrid catalysts, the hydrogenolysis and cleavage of methoxychlor occurred (Figure 6A).

Most of the variable-valence transition metals, such as Cu and Co ions, can replace Fe ions in the Fenton reaction [9]. Compared with Fe, Cu facilitates the interfacial electron transfer and, thus, accelerates the regeneration of the catalyst. Yan et al. [89] established a highly catalytic system for the degradation of ethylbenzene using Cu-based LDHs (CuMgFe-LDH) and H_2_O_2_. The degradation of ethylbenzene was achieved through the formation of active sites by surface-bound hydroxyl groups of LDHs and the generation of ^•^OH in the redox process of Cu(II)-Cu(III). In addition, due to the tunability of multi-metals in LDH laminates, LDHs with synergistic redox cycle of multiple transition metals can be designed to promote the regeneration of low-valent metal and improve the efficiency of the Fenton reaction. Wang et al. [90] developed a ternary CuNiFe-LDH to degrade phenol. Compared with NiFe-, CuFe-, and CuNiAl-LDHs, CuNiFe-LDH had better catalytic performance. In the conventional Fenton reaction process in which Fe^3+^ in the LDHs laminate was reduced to Fe^2+^ and then oxidized to Fe^3+^ again, Cu^+^ in the laminate could also be oxidized to Cu^2+^ to catalyze the Fenton reaction. Moreover, the metal–oxygen–metal bridge and highly dispersed NiO_6_ octahedra in LDHs allowed for a quick electron transfer and generated more ^•^OH radicles to degrade phenol to CO_2_ and H_2_O, and the mechanism of CuNiFe-LDH catalytic degradation of phenol is shown in Figure 6B.

Some substances, such as carbon-based materials, can also be loaded on LDHs to form stable and efficient Fenton catalysts. For example, the trimetallic LDH of CuMgAl-LDH combined with reduced graphene oxide (rGO) exhibited a triphasic synergistic effect with enhanced catalytic performance [91]. The structure of the catalyst improved the stability and electronic conductivity. Peng et al. [92] prepared CuAl-LDH for the degradation of ammonia nitrogen and found that CuAl-LDH aggregated easily. The addition of carbon fiber (CF) prevented the aggregation of the delaminated LDH nanosheets. The conductivity and porous structure of CF also accelerated the diffusion of ammonia nitrogen, thus improving the catalytic performance of CuAl/CF-LDH. The large specific surface area, abundant active sites, and strong chemical forces were involved in the ammonia nitrogen degradation (Figure 6C). 

In the application of LDHs as catalysts in Fenton reactions, most studies focused on the effect of different cations in the lamellae of LDHs. Few studies considered the effects of interlayer anions, which also affect catalytic activity. Costa-Serge et al. [93] evaluated the effect of different interlayer anions in the boronic acid-based LDH (CuMgFe-B(OH)_4_) on the degradation of 5-fluorouracil. It was found that the intermolecular B-O and B-OH can induce changes in the surface charge density of CuMgFe-B(OH)_4_ and accelerate the reduction in Cu(II) and Fe(III), which favors the generation of reactive oxygen species. Figure 6D explains the possibility that the presence of boric acid leads to the partial reduction in iron and copper on the catalyst surface. The Lewis acid-base bond between LDH hydroxy-boron and oxygen was strengthened and improved the stabilization of the hydrogen bond between boric acid and CuMgFe-B(OH)_4_ surface.

### 4.2. Persulfate-Based AOPs

Among different AOPs, SO_4_^•−^ dominated AOPs not only overcome the limitations of conventional Fenton system in terms of narrow pH applicability, large use of H_2_O_2_, ferrous salts, and lots of sludge yield in practical applications but also have the advantages of strong oxidative capacity, long half-life of radicles, and strong mass transfer capacity [94]. It is noteworthy that LDHs have a great advantage in the effective activation of PMS due to their controllable lamellar structure [95].

**Figure 7:**
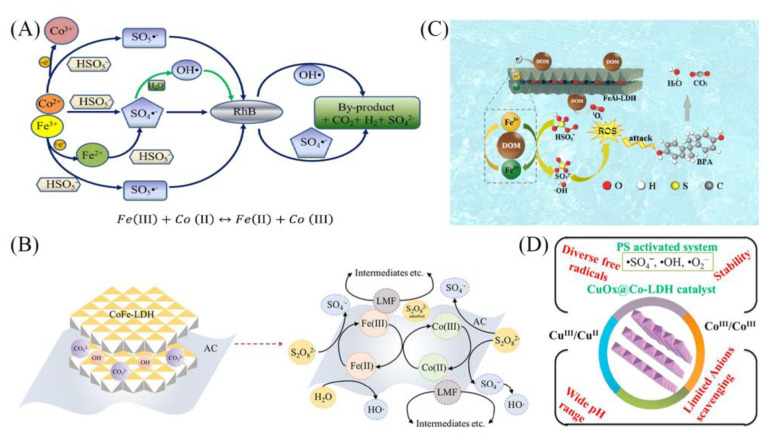
(**A**) Mechanism of RhB degradation in FeCo-LDH/PMS system [99]. (**B**) Activation of PS by AC@CoFe-LDH for lomefloxacin degradation [103]. (**C**) Removal mechanism of BPA in DOM-FeAl-LDH/PMS system [102]. (**D**) Advantages of CuOx@Co-LDH as an activator for PS [104]. Reprinted with permission from Elsevier.

Transition metals usually act as electron donors to activate PMS due to their advantages, such as low energy consumption and high efficiency [96]. Co^2+^, Ni^2+^, Fe^2+^, Cu^2+^, and Mn^2+^ have shown strong persulfate catalytic activity [97,98]. Gong et al. [99] prepared FeCo-LDH by coprecipitation method and used to degrade RhB by non-homogeneous activated PMS. In FeCo-LDH/PMS system, RhB was completely degraded within 10 min, and the removal efficiency reached more than 90%. As shown in Figure 7A, the redox process of Fe(II) and Co(II) generated electrons, activated PMS to produce reactive radicals, and the generated SO_4_^•−^ and ^•^OH simultaneously rapidly degraded RhB. The efficient regeneration of Co(II) may be the reason for the significant activation of PMS and the enhanced degradation of RhB. Li et al. [100] prepared CoFeLa-LDH catalyst by coprecipitation method, which exhibited excellent performance to activate PMS for tetracycline (TC) elimination. The degradation efficiency of TC in the CoFeLa-LDH_2_/PMS system was 90.1% within 10 min.

To solve the problem of metal leaching, it is proposed to prepare LDH-based composites by immobilizing LDHs onto a specific functional carrier. Carbon-based-LDHs composites with activated carbon (AC), GO, biochar, and various carbon polymer films as carriers can increase the degradation efficiency and stability compared to single LDHs catalysts [101,102,103]. Ma et al. [103] prepared the CoFe-LDH using the coprecipitation method, loaded it onto AC, and purified it with HCl to synthesize AC@CoFe-LDH. Compared with CoFe-LDH/PS system, the degradation efficiency of lomefloxacin by AC@CoFe-LDH was increased by 56% within 60 min, where SO_4_^•−^ played a major role in the degradation process. The catalyst itself could be regenerated in the redox reaction between Fe(II)/Fe(III) and Co(II)/Co(III), and the reaction of AC components with H_2_O could also restore the functional groups (Figure 7B). As dissolved organic matter (DOM) is of great interest as an important component of biochar, Ye et al. [102] obtained DOM-FeAl-LDH by incorporating DOM into FeAl-LDH and further used it to activate PMS to degrade BPA in water. Compared with FeAl-LDH/PMS system, the removal rate of BPA in DOM-FeAl-LDH/PMS system increased from 60% to 93% within 60 min. It was found that the catalyst activity and stability were improved after DOM incorporation. This was because the HA-like compounds in DOM could act as electron transfer mediators, which could accelerate the reduction in Fe(III). In addition, DOM itself could produce ROS, which reacts with dissolved oxygen and may lead to the production of ^1^O_2_ (Figure 7C). 

For LDH-based catalysts, some metal oxides or metal sulfides have been intensively utilized [104,105]. Ali et al. [104] dispersed Co-LDH in copper salt solution and calcined to obtain CuOx@Co-LDH and found that phenol was degraded within 40 min. The Cu^2+^ was detected in the solution, and no Co^2+^ was detected after the reaction. The degradation mechanism was mainly through the synergistic interaction between mixed valence metals to activate PS to produce SO_4_^•−^ and ^•^OH for phenol degradation (Figure 7D). The strong synergistic interaction between the active components in the LDH structure was the reason for the superior stability and activity of the CuOx@Co-LDH catalyst. Zeng et al. [105] synthesized hollow flower-like and rich oxygen vacancies of CoAl-LDH@CoSx catalyst and found that 98.5% of sulfamethoxazole (40 µmol/L) could be removed in 4 min, and the mineralization rate reached 20.4% in 6 min. Both results of quenching and radical trapping experiments showed that the main active substance in CoAl-LDH@CoSx/PMS system was ^1^O_2_.

### 4.3. Electrocatalytic System

The diversity of layer metal ions allows for the LDHs to have different physicochemical and electrical properties, which can be used as electrocatalysts in the wastewater treatment [106]. Currently, the most widely used oxygen evolution reaction (OER) catalysts in alkaline environments are commercial ruthenium and iridium oxides (RuO_2_, IrO_2_) [107], which cannot be used commercially on a large scale due to their high production costs and low electrochemical stability. It is well known that transition metals are abundant and have good catalytic properties for both OER and hydrogen evolution reactions (HER) [108]. Therefore, transition metal hydroxides are used to replace precious metal catalysts with great advantages, especially some composites containing multiple transition metals. Among them, Ni-based LDHs have become a subject of intensive materials in recent years [109]. Liu et al. [110] synthesized a series of Cr, Mn, and Co-doped NiFe-LDHs via the hydrothermal method, all of which enhanced the catalytic activity of OER in an alkaline medium. The improved catalytic performance could be attributed to the tuning of the electronic states of Ni and Fe atoms by the doped metal atoms. However, the stability of transition metal catalysts needs to be further improved compared to precious metals.

Non-precious metal catalysts are in full swing. For example, carbon nanotubes and graphene, which are rich in functional groups (−COOH, −OH, etc.) on their surfaces and have strong electrical conductivity, large specific surface area, and good thermal stability, and have attracted much attention in the field of electrocatalysis [111]. Xu et al. [112] prepared the three-dimensional nanocomposites by assembling NiAl-LDHs nanosheets on GO. The structure was unique, and it increased the specific surface area. The specific capacitance could reach 1300 F g^−1^ at higher current densities. After 500 cycles at 15.30 A g^−1^, the specific capacitance remained at 91% compared to 74% for pure NiAl-LDH. Meanwhile, the good stability of non-metallic materials under acidic and alkaline conditions makes them the preferred materials for electrocatalysts under high currents. However, the lower electronic conductivity hinders the further development of LDHs as high-performance electrocatalysts. To solve this problem, Duan et al. [113] prepared the NiFe-LDH/CNTs composites with alternating stacks of NiFe-LDH and CNTs layers. This unique structure provided ample active sites and efficient charge/electron transfer channels for OER electrocatalysis. The optimized composite required only 234 mV to achieve a current density of 10 mA cm^−2^ and exhibited better OER activity than RuO_2_. The excellent performance should be attributed to the synergistic effect of active NiFe-LDH with conductive CNTs and an intercalation structure with a highly accessible surface area. To meet the growing demand for electrochemical conversion systems, LDH electrocatalysts should focus more on compact design, low cost, and versatility.

## 5. Conclusions and Prospect

We reviewed the main research advances in LDH-based materials for wastewater treatment in recent years. The preparation approaches of modified LDHs mainly include coprecipitation, hydrothermal, ion exchange, calcination recovery, and sol–gel methods. Wastewater treatment methods using LDHs mainly involve adsorption, Fenton reaction, persulfate-based AOPs, and electrocatalytic method. The advantages of LDHs as adsorbents and catalysts, as well as the reaction mechanism, were mainly reviewed. 

Although the modification of LDHs and the removal of contaminants from water are studied, some shortcomings still remain and need to be further investigated.

(1) In terms of the preparation of LDHs, the method of high-performance LDHs is relatively cumbersome and not conducive to industrial production, which limits the application. In addition, LDHs are mostly in powder form and are difficult to be recovered after the treatment process;
(2) In terms of wastewater treatment using LDH-based materials, most of the studies are still on the laboratory scale. LDHs are often used to remove a single pollutant, while various pollutants usually coexist in the water environment;
(3) In terms of mechanism research, there is a lack of in-depth methods, and the quantitative contribution of each mechanism to the total capacity needs to be further investigated.

In view of the shortcomings of LDHs, we can put forward the challenges to be faced in future work:

(1) Due to the urgent demand for high-efficient and low-cost materials for treating wastewater, there is a need to produce sustainable, low-cost, and industry-scale LDHs. For better recovery of LDHs and improvement of reusability during large-scale use, magnetic or non-powdered LDHs can be designed and synthesized for wastewater treatment;
(2) To make full use of the advantages of LDHs and other nanomaterials, high-performance LDH-based composites can be prepared via various methods. For example, LDHs with multivariate ions and the combination of LDHs with other high-performance materials may be one of the research directions to obtain high-performance catalysts;
(3) More work from laboratory to industrial production is urgently needed. There is also a need to strengthen the research on the performance of modified LDHs in removing specific pollutants selectively from multiple pollutants;
(4) Further in-depth studies on the mechanisms of LDHs for various pollutants can be carried out by means of spectroscopic characterization, interfacial chemical methods, and theoretical calculations. The contribution of each mechanism should be conducted to understand the interfacial process.

## Data Availability

Not applicable.
